# miR-96-5p targets PTEN expression affecting radio-chemosensitivity of HNSCC cells

**DOI:** 10.1186/s13046-019-1119-x

**Published:** 2019-03-29

**Authors:** Mahrou Vahabi, Claudio Pulito, Andrea Sacconi, Sara Donzelli, Marco D’Andrea, Valentina Manciocco, Raul Pellini, Paola Paci, Giuseppe Sanguineti, Lidia Strigari, Giuseppe Spriano, Paola Muti, Pier Paolo Pandolfi, Sabrina Strano, Shahrokh Safarian, Federica Ganci, Giovanni Blandino

**Affiliations:** 1 Oncogenomics and Epigenetics Unit, IRCCS-Regina Elena National Cancer Institute, 00144 Rome, Italy; 2 Cell and Molecular Biology Department, School of Biology, College of Science, University of Tehran, Tehran, 1417614411 Iran; 3 Unit of Radiotherapy, IRCCS-Regina Elena National Cancer Institute, Rome, Italy; 4 Unit of Otolaryngology, IRCCS-Regina Elena National Cancer Institute, Rome, Italy; 5 Institute for Systems Analysis and Computer Science “A. Ruberti”, National Research Council, Rome, Italy; 6 SysBio Centre for Systems Biology, Rome, Italy; 7 Laboratory of Medical Physics and Expert Systems, IRCCS-Regina Elena National Cancer Institute, Rome, Italy; 8 Unit of Otorhinolaryngology, Humanitas University, Milan, Italy; 9 Department of Oncology, Juravinski Cancer Center, McMaster University, Hamilton, Canada; 10 Cancer Research Institute, Beth Israel Deaconess Cancer Center, Department of Medicine and Pathology, Harvard Medical School, Boston, MA USA

**Keywords:** miRNAs, TP53 mutations, miR-96-5p, Head and neck cancer, Local recurrence, Migration, Radiotherapy, Chemotherapy, PTEN, PI3K-Akt signalling pathway

## Abstract

**Background:**

Head and neck squamous cell carcinoma (HNSCC) is the sixth leading cancer worldwide. They are typically characterized by a high incidence of local recurrence, which is the most common cause of death in HNSCC patients. *TP53* is the most frequently mutated gene in HNSCC and patients carrying *TP53* mutations are associated with a higher probability to develop local recurrence. MiRNAs, which are among the mediators of the oncogenic activity of mt-p53 protein, emerge as an appealing tool for screening, diagnosis and prognosis of cancer. We previously identified a signature of 12 miRNAs whose aberrant expression associated with TP53 mutations and was prognostic for HNSCC. Among them miR-96-5p emerges as an oncogenic miRNAs with prognostic significance in HNSCC.

**Methods:**

To evaluate the oncogenic role of miR-96-5p in a tumoral context, we performed colony formation, cell migration and cell viability assays in two HNSCC cell lines transfected for miR-96-5p mimic or inhibitor and treated with or without radio/chemo-therapy. In addition, to identify genes positively and negatively correlated to miR-96-5p expression in HNSCC, we analyzed the correlation between gene expression and miR-96-5p level in the subset of TCGA HNSCC tumors carrying missense *TP53* mutations by Spearman and Pearson correlation. To finally identify targets of miR-96-5p, we used in silico analysis and the luciferase reporter assay to confirm PTEN as direct target.

**Results:**

Our data showed that overexpression of miR-96-5p led to increased cell migration and radio-resistance, chemotherapy resistance in HNSCC cells. In agreement with these results, among the most statistically significant pathways in which miR-96-5p is involved, are focal Adhesion, extracellular matrix organization and PI3K-Akt-mTOR-signaling pathway. As a direct target of miR-96-5p, we identified PTEN, the main negative regulator of PI3K-Akt signalling pathway activation.

**Conclusions:**

These results highlight a new mechanism of chemo/radio-resistance insurgence in HNSCC cells and support the possibility that miR-96-5p expression could be used as a novel promising biomarker to predict radiotherapy response and local recurrence development in HNSCC patients. In addition, the identification of pathways in which miR-96-5p is involved could contribute to develop new therapeutic strategies to overcome radio-resistance.

**Electronic supplementary material:**

The online version of this article (10.1186/s13046-019-1119-x) contains supplementary material, which is available to authorized users.

## Background

Head and neck cancers are the sixth leading cancer worldwide and they are represented mainly by the squamous cell carcinoma (HNSCC) occurring in the oral cavity, pharynx, or larynx [1–4].

They are typically characterized by a high incidence of local recurrences, which are the most common cause of death in HNSCC patients, taking place in 60% of cases [5, 6]. The current standard therapies are surgical and medical treatment followed by adjuvant radiotherapy (RT) with or without chemotherapy. Unfortunately, advances in treatments for HNSCC over the past two decades have not improved overall disease outcomes [7, 8] and radio and chemo-resistance (intrinsic or acquired) remain one of the major challenges in the current therapy of HNSCC.

Additionally, the TNM staging system, used to classify HNSCC patients, does not adequately address the molecular heterogeneity of HNSCC tumors and patients with the same TNM stages have a heterogeneous response to therapy [9]. This indicates the need to obtain a more detailed molecular characterization in order to improve our understanding of radio and chemo-resistance mechanisms, to identify biomarkers for early detection of local recurrence and treatment response. However, although several molecules can potentially predict response to therapy and clinical outcome in HNSCC, to date, no biomarkers have been definitively introduced in the clinical practice. Mutation in *TP53* tumour suppressor gene is the most frequently detectable genetic alteration (about 70–80%) reported in HNSCC [10, 11]. Several evidences show that mutant p53 protein is one of the main players involved in radio/chemo-resistance insurgence and it generally predicts poor outcome and treatment failure in HNSCC patients [12–15]. In addition to *TP53* gene, among the best promising biomarkers, miRNAs, are considered as an appealing tool for screening, diagnosis and prognosis of cancer [16–19]. miRNAs are small non-coding RNA (17–22 nucleotides) which function as post transcriptional regulators of target gene expression through interaction with mainly 3’UTR of target mRNAs [20]. The deregulation of miRNA expression with oncogenic or tumor suppressor function in several diseases, including HNSCC cancer, has been reported [19, 21]. One of the emerging miRNAs as oncogene and biomarker in HNSCC is miR-96-5p [22, 23].

In our previous studies, we demonstrated that the expression of miR-96-5p is associated to *TP53* status and its high expression level, individually and in combination with other miRNAs, was able to predict local recurrence independently from other clinical co-variables either in tumors or in histologically tumor-free peritumoral tissue [14, 15, 24].

In this study, we aim at deeply characterizing the oncogenic activity of miR-96-5p in HNSCC cells carrying mutant *TP53* gene, focusing the attention in particular on its role in radio/chemo-resistance, for which no evidences are present.

We demonstrate that miR-96-5p is up-regulated in tumor versus normal tissues in two different HNSCC cohorts of patients and we confirm that this up-regulation is significantly stronger in patients carrying *TP53* mutations than the wild type group. Next, we show that overexpression of miR-96-5p in the HNSCC cells carrying mutant p53 protein leads to increased cell migration, and, finally, we provide the first evidence that miR-96-5p is involved in radio- and chemo-therapy resistance, at least in part, by directly targeting PTEN mRNA and maintaining aberrantly activated the PI3K-AKT pathway.

## Materials and methods

### Cell lines and culture conditions

Cal 27, FaDu and H1299 cell lines were obtained from ATCC (Rockville, MD, USA). These cells were cultured in RPMI-1640 medium (Invitrogen-GIBCO, Carlsbad, CA) supplemented with 10% fetal bovine serum, penicillin (100 U/mL), and streptomycin (100 mg/mL; Invitrogen-GIBCO). All cell lines were grown at 37 °C in a balanced air humidified incubator with 5% CO2. All cell lines were tested by PCR/IF for Mycoplasma presence.

### Cell transfection

mirVana™ miRNA mimic negative control #1 (Ambion) or hsa-miR-96-5p mirVana™ miRNA mimic (Ambion # 4464066) at final concentration of 5 nM for over expression of miR-96-5p and mirVana™ miRNA inhibitor negative control #1 or has-miR-96-5p mirVana™ miRNA inhibitor (Ambion # 4464084) at final concentration of 10 nM for depletion the miR-96-5p expression were transfected into HNSCC cell lines (Cal 27, FaDu) using Lipofectamine RNAiMAX (Invitrogen) according to the manufacturer’s instructions. To decrease PTEN expression, a siRNA PTEN (5′ GCUACCUGUUAAAGAAUCA 3′ and, siRNA SCR 5′ CUAUAACGGCGCUCGAUAUTT 3′, eurofins genomics) were used and transfected with RNAiMAX into the HNSCC cells.

### RNA extraction and microRNA expression analysis

Total RNA was extracted using the TRIZOL Reagents (GIBCO) following the manufacturer’s instructions. The concentration and purity of total RNA was assessed using a Nanodrop TM1000 spectrophotometer (Nanodrop Technologies, Wilmington, DE, USA). 30 ng of total RNA was reverse transcribed using the TaqMan microRNA Reverse Transcription Kit (Thermo Fisher Scientific). Real time-PCR of miRNA expression was carried out in a final volume of 10 ul using ABI Prism 7000 Sequence Detection System (Thermo Fisher Scientific). The PCR Reactions were initiated with a 10 min incubation at 95 °C followed by 40 cycles of 95 °C for 15 s and 60 °C for 60s. qRT-PCR quantification of miRNA expression was performed using TaqMan MicroRNA® Assays (Thermo Fisher Scientific) according to the manufacturer’s protocol. RNU44 and RNU48 were used as endogenous control to normalize miRNA expression. All reactions were performed in triplicate.

### Cell viability assay

Viability of treated cells was assessed using ATPlite assay (Perkin Elmer, Massachusetts, USA) at the indicated time points, accordingly to the manufacturer’s instructions. Cells (8 × 10^2 cells) were seeded in 96 well-plates and cultured for 24–48–72-96 h. Each plate was evaluated immediately on a microplate reader (Expire Technology, Perkin Elmer).

### Dual luciferase reporter assay

Wild type 3’UTR of PTEN was cloned in to the psi-CHECK2 vector (a kind gift from Dr. Pier Paolo Pandolfi) Next, H1299 cells were co-transfected in 24-well dishes using Lipofectamine 2000 (Invitrogen) with 100 ng of PTEN-3′-UTR (wt)-Luciferase vectors (psiCHECK-2, Promega), 100 ng of PTEN-CDS-psi-CHECK2 vector and 20 nM mir-Vana™ miRNA Mimic Negative Control #1 (Ambion) or hsa-miR-96-5p mirVana™ miRNA Mimic (Ambion # 4464066). Cells were harvested 48 h post-transfection and luciferase activities were analyzed by the dual-luciferase reporter assay system (Promega, Madison, WI) in the GloMax 96 Microplate Luminometer (Promega). Each sample was transfected in duplicate. Each experiment was repeated in triplicate.

### Lysate preparation and immunoblotting analysis

Cells were lysed in buffer with 50 mM Tris–HCl pH 8, with 1% NP-40 (IgepalAC-630) 150 mM NaCl, 5 mM EDTA and fresh protease inhibitors. Extracts were sonicated for 10 s and centrifuged at 12000× rpm for 10 min to remove cell debris. Protein concentrations were determined by colorimetric assay (Bio-Rad). Western blotting was performed using the following primary antibodies: mouse monoclonal anti-Nucleolin (Abcam # ab13541), rabbit monoclonal anti-PTEN (Cell Signaling # 9552), rabbit monoclonal anti-AKT (Cell Signaling # 4685), rabbit monoclonal anti-P-AKT serin473 (Cell Signaling #4058). Secondary antibodies used were goat anti-mouse and goat anti-rabbit, conjugated to horseradish peroxidase (Amersham Biosciences,Piscataway, NJ). Immunostained bands were detected by chemiluminescent method (Pierce, Rockford, IL).

### Transwell migration assay

Migration assay was performed using a 24-well plate with a non-coated 8-mm pore size filter in the insert chamber (Falcon). Cells were transfected with mirVana™ miRNA Mimic Negative Control #1 (Ambion) or hsa-miR-96-5p mirVana™ miRNA Mimic (Ambion # 4464066), for miR-96-5p depletion we used mirVana™ miRNA Inhibitor Negative Control #1 or hsa-miR-96-5p mir- Vana™ miRNA Inhibitor (Ambion # 4464084) at final concentration of 10 nM. For PTEN depletion, we used 0.1 uM siSCR or siPTEN (Eurofins genomics). 48 h after transfection, HNSCC cells were resuspended in RPMI media with 1% FBS and seeded into the insert chamber. Cal 27 and FaDu cells were allowed to migrate for 48 h or 72 h into the bottom chamber containing 0.7 ml RPMI media containing 10% FBS in a humidified incubator at 37 °C in 5% CO2. Migrated cells that attached to the outside of the filter were visualized by staining with DAPI and counted.

### Clonogenic assays

Cal 27 and FaDu cell lines were transfected with mirVana™ miRNA mimic negative control #1 (Ambion) or hsa-miR-96-5p mirVana™ miRNA mimic (Ambion # 4464066) and mirVana™ miRNA inhibitor negative control #1 or has-miR-96-5p mirVana™ miRNA inhibitor (Ambion # 4464084). 48 h after transfection, cells were detached and seeded at 500–1500 cells/well into 6-well dishes (Falcon) in drug-free media. Fresh media (25%) was added every three days. Colonies were stained with crystal violet and colonies (> 50 cells) counted after 10–14 days.

### Bioinformatic analysis of TCGA dataset

As validation set, we used TCGA head and neck squamous cell carcinoma (TCGA Research Network: http://cancergenome.nih.gov/). Normalized TCGA HNSC data were obtained from http://gdac.broadinstitute.org/ (10.7908/C11G0KM9).

miRNA deregulation was assessed by paired or unpaired Student’s t-test. Significance was defined at the *P* < 0.05 level.

### Kaplan–Meier analysis

Local recurrence-free survival was evaluated using Kaplan–Meier analysis and Cox proportional hazard regression model. Intensity levels of tumoral samples were divided in three subgroups and survival analysis was conducted by comparing the highest values with the medium and low subgroups. The log-rank test was used to evaluate differences between curves. Significance was defined at the P < 0.05 level. All the analyses were performed by MATLAB (The MathWorks).

### In silico miRNA targets identification

Several prediction target tools were examined using the web server tool MirWalk3 (http://zmf.umm.uni-heidelberg.de/apps/zmf/mirwalk2/).

Given a list of microRNAs, we downloaded the experimentally validated miRNA-target interactions from miRTarBase web site (Release 7.0, September 2017). Then, we built a network of miRNA-target interactions by using R programming language (Release 3.4.4, March 2018) for network visualization and analysis [25].

### Pathway enrichment analysis

Pathway enrichment analysis was performed using the program http://mirwalk.umm.uni-heidelberg.de/. Positively and negatively correlated genes to miR-96-5p expression from TCGA dataset were selected and used for the program as explained in the relative paragraph of the results (Additional file 1: Table S1). For the cut-off, in the program the following criteria were used: minimum overlap with input list: 10 genes and *P* < 0.05.

## Results

### miR-96-5p upregulation correlates with *TP53* status and predicts local recurrence development in HNSCC patients

In our previous studies, we have identified TP53-mutation associated alterations of miRNAs expression in HNSCC tissues [14, 15, 24]. In particular, we demonstrated that the expression of a specific *TP53*-mutation associated four-miRNAs signature predicts local recurrence insurgence in tumors and matched histologically tumor-free peritumor tissues from HNSCC patients [14, 24]. miR-96-5p was included in this signature and because its oncogenic activity in HNSCC cells has not been deeply characterized yet, here we decided to focus our attention on this miRNA.

**Fig. 1:**
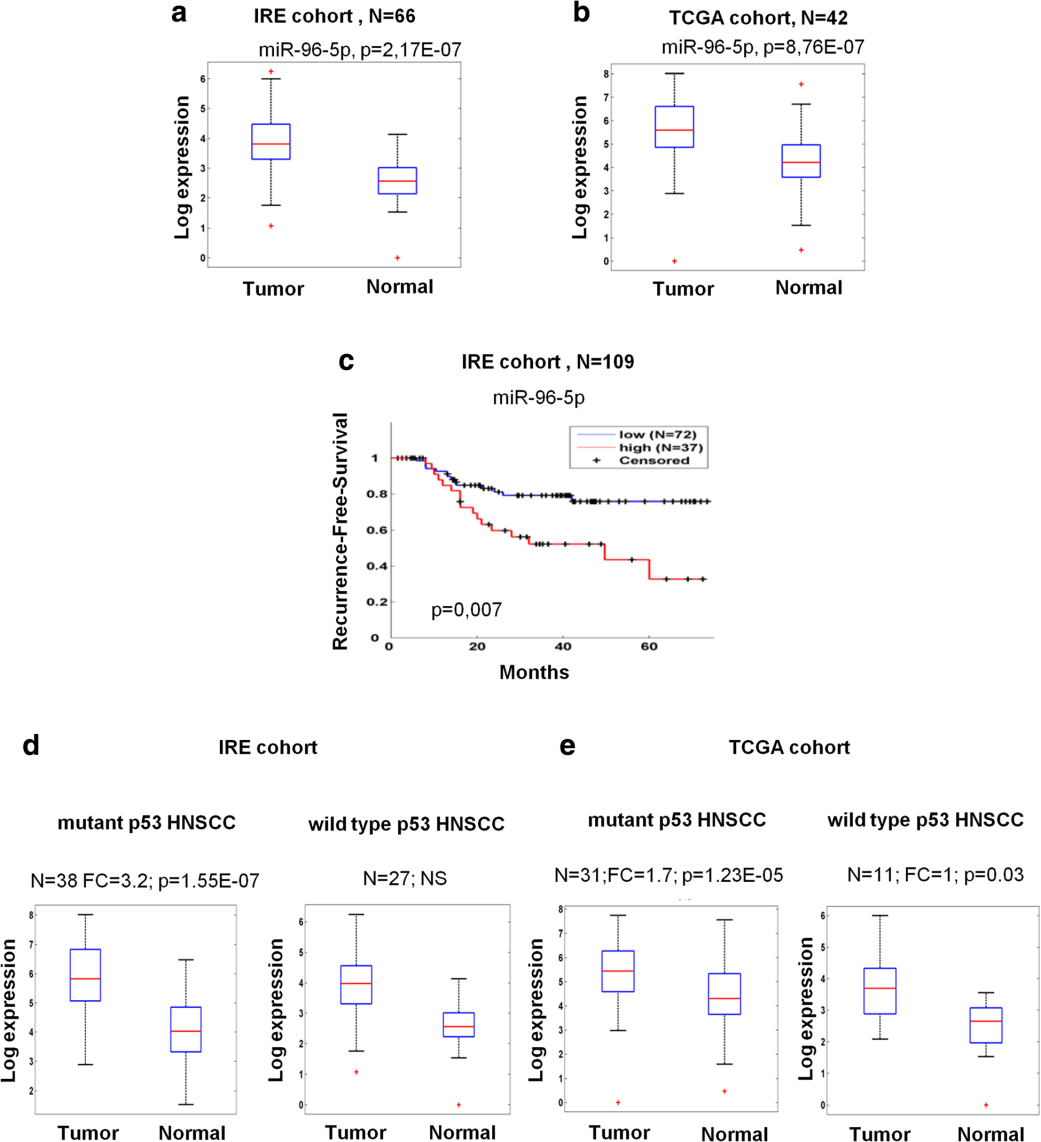
miR-96-5p upregulation is stronger in *TP53* mutated HNSCC and predicts local recurrence in HNSCC patients. **a** Box plot showing expression level of miR-96-5p in the tumoral and normal tissues from IRE cohort. **b** Box plot of the expression level of miR-96-5p in the tumoral and normal tissues from TCGA cohort. **c** Kaplan–Meier (KM) analysis showing correlation between the expression level of miR-96-5p and recurrence-free survival in the HNSCC patients from IRE cohort. **d-e** Comparison of miR-96-5p expression between *TP53* mutated and wild type tumors from matched samples IRE cohort (**d**) and from matched samples TCGA cohort (**e**). N: number of samples, FC: Fold change, p: *p*-value

We first evaluated the expression level of the miR-96-5p in matched tumoral and normal tissues collection from head and neck squamous cell carcinoma patients of IRE and TCGA cohorts (Fig. 1a-b).

The expression level of miR-96-5p was significantly higher in the tumoral tissues in comparison with the non-tumoral tissues from 66 matched samples of IRE cohort (Fig. 1a) and from 42 matched samples of TCGA cohort (Fig. 1b). Additionally, as shown in Fig. 1c and in agreement with our previous data [14, 15], we showed that high expression of miR-96-5p predicts local recurrence development in HNSCC patients by Kaplan-Meier (KM) analysis. Next, to further confirm that the expression level of this miRNA is associated to *TP53* status, we evaluated its expression level in the matched subgroup tumor versus normal tissues of HNSCC patients carrying *TP53* mutations versus the *TP53* wild type. As shown in Fig. 1d-e, we found that its up-regulation in tumors versus non-tumoral tissues was significantly stronger in the subset of mutant p53 patients. As mutation in *TP53* tumor suppressor gene is the most frequently detectable genetic alterations (about 70–80%) reported in HNSCC [10], we decided to specifically assess the miR-96-5p oncogenic activity TP53 mutated context HNSCC cell lines.

### Overexpression of miR-96-5p promotes cell migration, but not cell proliferation in HNSCC cells

In order to investigate the miR-96-5p oncogenic activity TP53 mutated context, we performed a series of experiments to evaluate migration, survival, proliferation, clonogenicity and sensitivity to adjuvant therapy in two selected HNSCC cell lines, Cal 27 and FaDu [26].

**Fig. 2:**
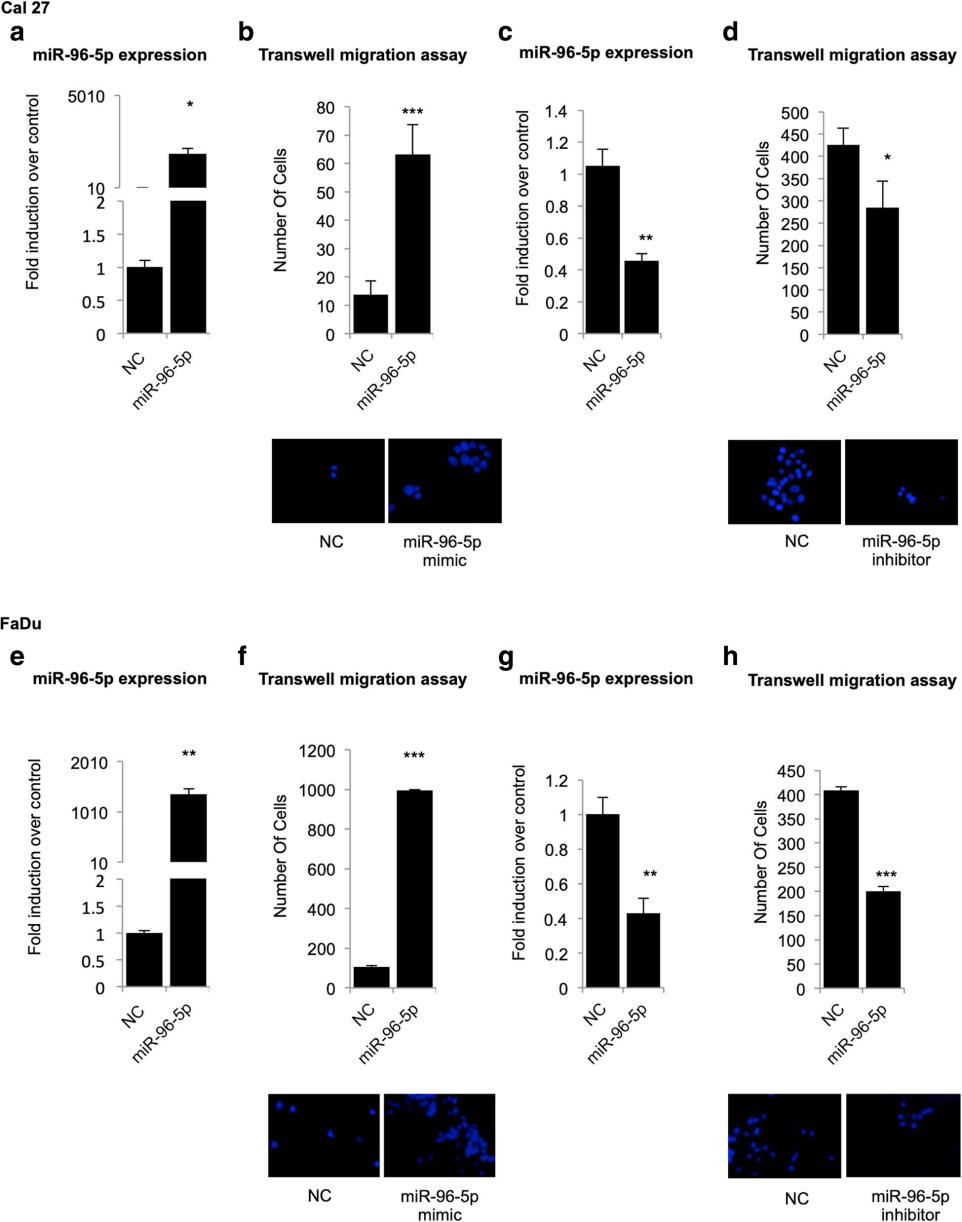
High expression of miR-96-5p promotes cell migration in HNSCC cell lines carrying mutant p53 protein. Two HNSCC cell lines (Cal 27, FaDu) were transfected with miR-96-5p mimic and inhibitor. The efficiency of transfection was evaluated by RT-qPCR in Cal 27 (**a-c**) and FaDu (**e-g**) cells. **b, d, f, h** Representative micrographs of transwell migration in Cal 27 (**b-d**) and FaDu (**f-h**) cell lines, DAPI staining was used to quantify the migrated cells on the outer surface of the filter. Histogram shows the number of migrated cells after using miR-96-5p mimic (**b, f**) and miR-96-5p inhibitor (**d, h**) in the Cal 27 and FaDu cell lines. Bars show the means of at least two experiments performed in triplicate. The *p*-value refers to matched control versus miR-96-5p mimic and miR-96-5p inhibitor transfected samples.*P < 0.05; **P < 0.001; ***P < 0.0001. NC: Negative control

First, we assessed the influence of miR-96-5p on cell migration, by transfection of the miR-96-5p mimic, miR-96-5p inhibitor or relative negative control into Cal 27 and FaDu cell lines. The efficiency of miR-96-5p mimic and inhibitor transfection on HNSCC cells was evaluated by RT-qPCR on Cal 27 (Fig. 2a, c) and FaDu (Fig. 2e, g) cells. The effect of miR-96-5p on HNSCC cell migration was analyzed by Transwell migration assay and the migratory ability of the transfected cells was measured and compared with that of negative control (NC) at 72 and 96 h post-transfection. As shown in Fig. 2b and f, overexpression of miR-96-5p, significantly increased cell migration of both cell lines. Conversely, down-regulation of miR-96-5p inhibited the cell migration of Cal 27 (Fig. 2d) and FaDu (Fig. 2h) cells. We next evaluated the impact of miR-96-5p on cell proliferation and clonogenicity by performing clonogenic assay, cell cycle analysis and growth curve. Collectively, the results showed that miR-96-5p did not affect cell proliferation but did affect cell migration. (Additional file 2: Supplementary material and methods and Additional file 3: Figure S1).

### miR-96-5p expression enhances resistance to radiotherapy and cisplatin-based chemotherapy in HNSCC cells

**Fig. 3:**
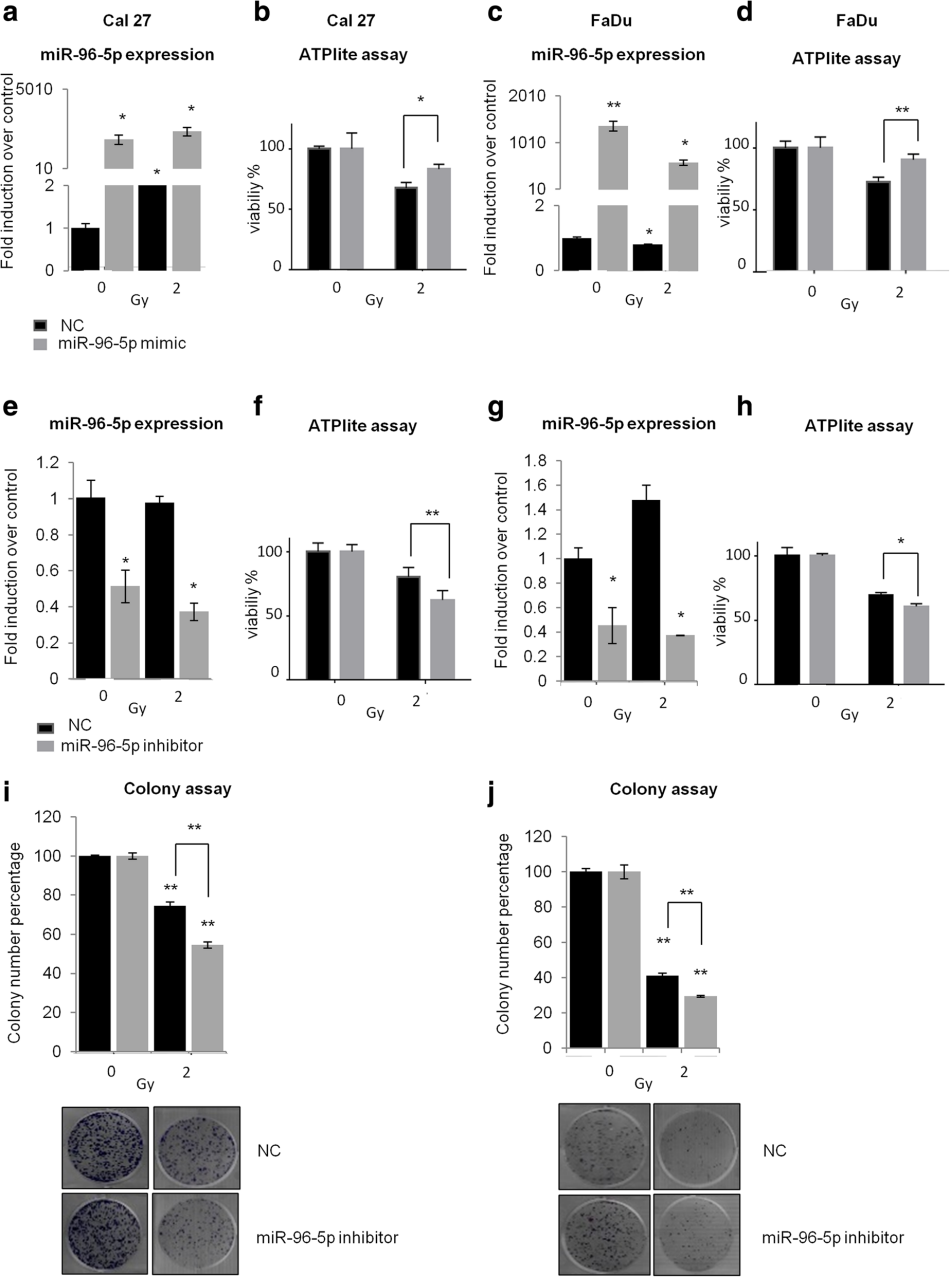
miR-96-5p expression affects radiosensitivity of Cal 27 and FaDu cells. Cal 27 and FaDu cells were transfected with miR-96-5p mimic and inhibitor. After 48 h, cells were treated with 2Gy dose of irradiation. **a-d** RT-qPCR analysis showing efficiency of miR-96-5p mimic transfection in Cal 27 cells (**a**) and in FaDu cells (**c**). Cell viability after transfection and radiation of Cal 27 (**b**) and FaDu (**c**) cells has been assessed by ATPlite assay. **e-h** RT-qPCR analysis showing efficiency of miR-96-5p inhibitor transfection in Cal 27 cells (**e**) and in FaDu (**g**) cells. Cell viability after transfection and radiation of Cal 27 (**f**) and FaDu (**h**) cells has been assessed by ATPlite assay. **i-j** Colony formation assay of Cal 27 (**i**) and FaDu (**j**) cells after transfection with miR-96-5p inhibitor and radiation treatment at 2Gy dose. Histogram bars show the means of at least three experiments performed in triplicate. *P < 0.05; **P < 0.001. NC: Negative control

Radio and chemo-resistance is a critical issue in HNSCC treatment and is frequently related to local recurrence development [7, 27]. To this purpose we investigated the effect of miR-96-5p expression on radio- and chemo-sensitivity by performing cell viability and clonogenic assays on HNSCC cell lines. At 48 h after transfection with miR-96-5p mimic or inhibitor, Cal 27 and FaDu cells were treated with 2Gy dose of irradiation and the cell viability was assessed by ATPlite assay. The efficiency of transfection was evaluated by RT-qPCR (Fig. 3a, c, e and g). As shown in Fig. 3b and d, overexpression of miR-96-5p increased cell radio-resistance respectively in Cal 27 and FaDu cell lines. On the contrary, down regulation of miR-96-5p enhanced sensitivity to irradiation by decreasing cell viability (Fig. 3f, h) and the number of colonies formed in the cells treated with 2Gy in comparison with the negative control (Fig. 3i, j).

**Fig. 4:**
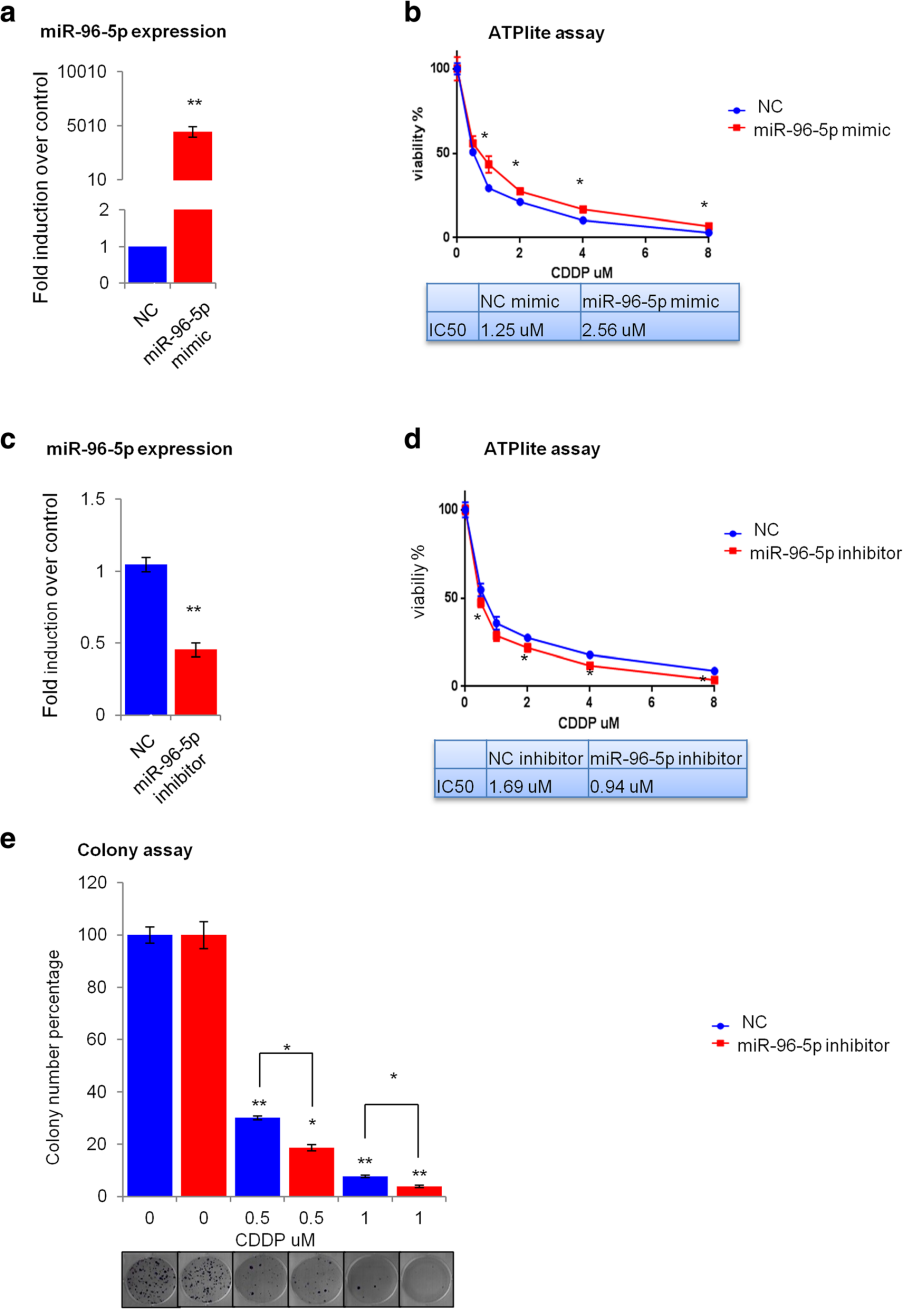
miR-96-5p expression affects the sensitivity of Cal 27 cells to chemotherapy. Cal 27 cells were transfected with miR-96-5p mimic and inhibitor. After 48 h cells were treated with different concentration of cisplatin (0.5,1,2,4,8 μg/ml). **a** RT-qPCR data showing efficiency of miR-96-5p mimic transfection. **b** Cell viability analysis of Cal 27 cells treated with miR-96-5p mimic and different concentrations of cisplatin in comparison with the negative control. **c** RT-qPCR data showing efficiency of miR-96-5p inhibitor transfection. **d** Cell viability analysis of Cal 27 cells treated with miR-96-5p inhibitor and different concentration of cisplatin in comparison with the negative control. **e** Colony formation assay performed on Cal 27 cells treated with miR-96-5p inhibitor and different concentration of cisplatin in comparison with the negative control. Histogram bars show the means of at least three experiments performed in triplicate.*P < 0.05; **P < 0.001. NC: Negative control, CDDP: Cisplatin

Next, we analyzed whether miR-96-5p expression could affect the sensitivity of HNSCC cells to cisplatin-based chemotherapy. After 48 h of transfection with miR-96-5p mimic or miR-96-5p inhibitor, Cal 27 and FaDu cells were treated with different concentrations of cisplatin (0.5, 1, 2, 4, 8 μg/ml) and the cell viability was assessed by ATPlite assay. The efficiency of transfection in Cal 27 (Fig. 4a, c) and FaDu (Additional file 4: Figure S2a, S2c) cells was detected by RT-qPCR. ATPlite assay indicated that overexpression of miR-96-5p decreased cell sensitivity to cisplatin by increasing cell viability in Cal 27 (Fig. 4b) and FaDu (Additional file 4: Figure S2b) cells. Indeed, changes in IC50 value were found in the transfected cells according to the presence of cisplatin treatment; overexpression of miR-96-5p led to an increase of IC50 value from 1.252 uM to 2.560 uM in comparison with the negative control in Cal 27 cells (Fig. 4b) and from 4.182 uM to 6.568 uM in FaDu cells (Additional file 4: Figure S2b). Conversely, down regulation of miR-96-5p expression enhanced cell sensitivity to cisplatin by decreasing cell viability after cisplatin treatment in Cal 27 (Fig. 4d) and FaDu (Additional file 4: Figure S2d) cells. As shown in Fig. 4d, IC50 value changed from 1.690 uM to 0.9435 uM compared to negative control for Cal 27 and from 3.890 uM to 2.016 uM for FaDu (Additional file 4: Figure S2d) cell lines. The correlation between miR-96-5p expression level and chemo-sensitivity was also confirmed by clonogenicity assay. As shown in Fig. 4e for Cal 27 cells and Additional file 4: Figure S2e for FaDu cells, downregulation of miR-96-p5 expression caused a decrease of the colony formation ability in cells treated with 0.5 and 1 μg/ml concentration of cisplatin compared to the transfected cells not treated with cisplatin.

### Identification of mRNAs correlated to miR-96-5p in HNSCC tumors carrying mutant p53 protein

To explore the functional output of the miR-96-5p deregulation in HNSCC tissues specifically in a mutant p53 context, we combined the information of miRNA expression levels with the gene expression profiles of 176 HNSCC patients carrying missense p53 mutations from TCGA cohort. First, we excluded all the mRNAs that resulted correlated to miR-96-5p expression also in HNSCC subgroup carrying wild type p53 from TCGA cohort. Then, we selected only the genes positively or negatively correlated with miR-96-5p according to a Pearson and Spearman’s correlation coefficient higher than 0.3 in absolute value predicted by MirWalk3. We obtained 214 mRNAs, of which 58 positively and 156 negatively correlated to the expression of miR-96-5p in tumor tissues of HNSCC patients from TCGA cohort (Additional file 1: Table S1).

Functional classification of these genes showed enrichment for genes belonging to various cancer-related pathways; many of them are in agreement with the miR-96-5p biological role related to the cell migration and resistance to adjuvant therapy demonstrated in HNSCC cells, as the focal Adhesion, extracellular matrix organization and PI3K-Akt-mTOR-signaling pathway (Table 1). Among the most significant predicted pathway, we found the PI3K-Akt pathway, which has emerged as one of the most frequently altered in cancer including HNSCC [28, 29]. This pathway plays a crucial role in regulating a broad range of cellular functions including cell growth, death, adhesion and migration, chemo-resistance, and radio-resistance in cancer cells [30]. One of the main inhibitor of the PI3K/AKT/mTOR pathway is PTEN, which is included in the list of mRNAs negatively correlated to miR-96-5p expression (Additional file 1: Table S1 sheet 1).

**Table 1:** Pathways enrichment analysis of genes correlated to miR-96-5p in a mutant *TP53* context

Pathway	*p*-value
Focal adhesion - *Homo sapiens* (human)	2.10E-05
Focal Adhesion	3.02E-05
Signaling by Receptor Tyrosine Kinases	3.29E-05
Regulation of actin cytoskeleton- Homo sapiens (human)	0.00016
Extracellular matrix organization	0.000163
PI3K-Akt signaling pathway - Homo sapiens (human)	0.000829
Cytokine-cytokine receptor interaction - Homo sapiens (human)	0.001243
Signal Transduction	0.001871
PI3K-Akt Signaling Pathway	0.002061
Human papillomavirus infection - Homo sapiens (human)	0.004174
Pathways in cancer - Homo sapiens (human)	0.008498
Axon guidance	0.008829
Hemostasis	0.023744
Post-translational protein modification	0.028931

P < 0.05 was considered significant

### PTEN is a direct target of miR-96-5p

**Fig. 5:**
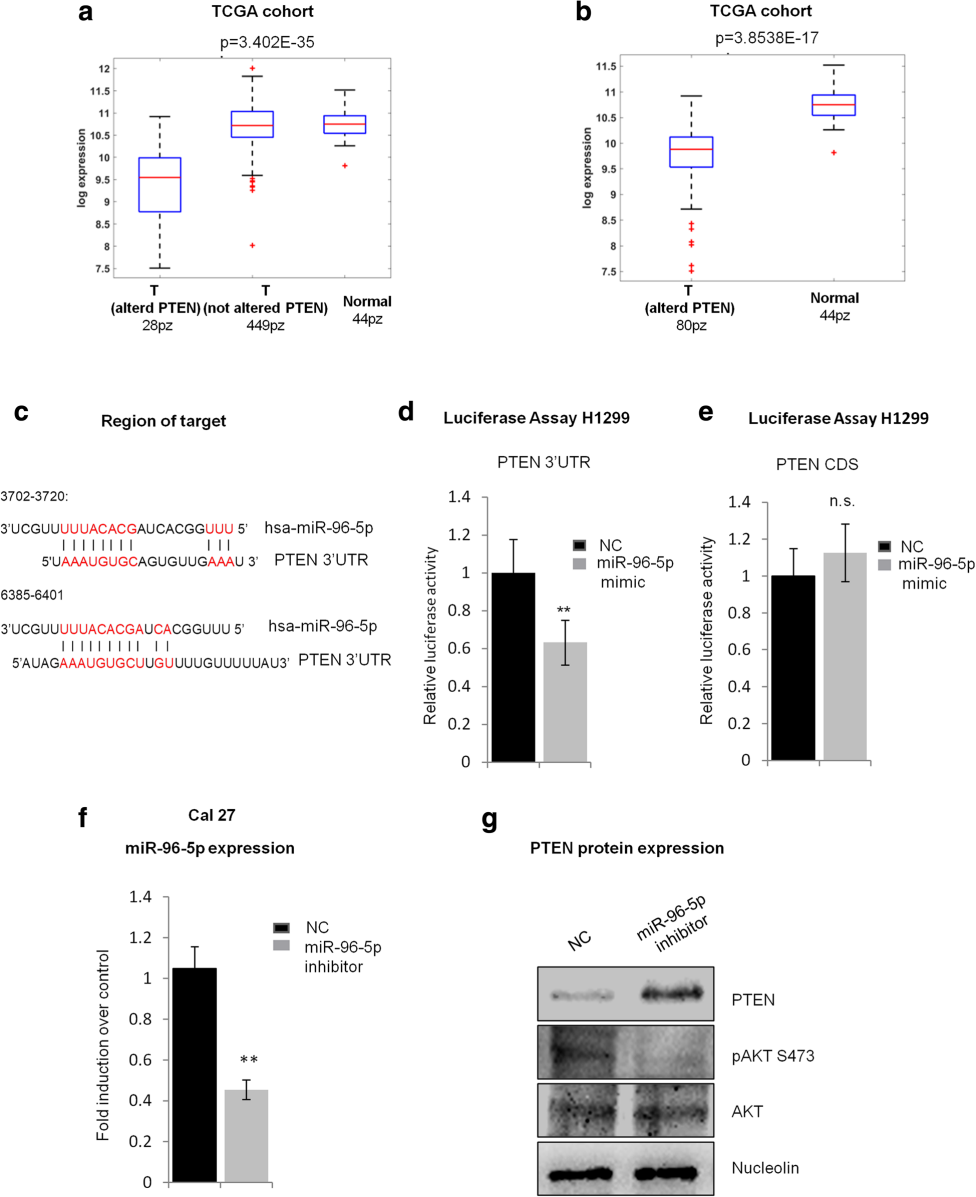
miR-96-5p targets PTEN and affects PI3K activation. **a** PTEN distribution in tumoral samples with altered PTEN (mutated or deleted), with not altered PTEN and in normal samples. **b** PTEN distribution in tumoral samples with altered PTEN or with down-regulation of PTEN higher than 0.5 log2 folds and in normal samples. **c** Position of the miR-96-5p target site in the 3’UTR of PTEN predicted by miRwalk 3.0. **d** Luciferase activity of PTEN-3’UTR-WT reporter gene in H1299 cell line. Cells were transiently transfected with miR-96-5p mimic or control mimic. The histogram bars show the means of three experiments performed in duplicate, **P < 0.001. **e** Luciferase activity of PTEN-CDS-WT reporter gene in H1299 cell line which were transiently transfected with miR-96-5p mimic or control mimic. Bars show the means of two experiments performed in triplicate. **f** RT-qPCR graph showing miR-96-5p expression level in Cal 27 cell line after miR-96-5p inhibitor transfection, **P < 0.001. **g** Western-blot analysis of PTEN, p-AKT and AKT protein expression level in Cal 27 cells that were transfected with miR-96-5p inhibitor or control inhibitor. NC: Negative control, T: tumor

As described above, among the genes predicted as putative targets of miR-96-5p (Additional file 1: Table S1 sheet 1), we identified PTEN. By interrogating HNSCC TGCA we found that PTEN expression is reduced in almost 15% of the patients. This is due either genetic alterations such as mutations and deletions or yet undiscovered epigenetic mechanisms that might also include miRNA targeting (Fig. 5a, b) [31]. To validate whether the PTEN mRNA is a direct target of miR-96-5p through the binding to its cognate site on the 3’UTR of PTEN (accordingly to MirWalk3 Fig. 5c), we performed a luciferase reporter assay. Wild type 3’UTR of PTEN cloned into the psi-CHECK2 vector was co-transfected with miR-96-5p mimic or control mimic into the H1299 cells. As shown in Fig. 5d, we found that miR-96-5p significantly reduced the relative luciferase activity of the wild-type reporters. Next, we checked the activity of these reporters when we co-transfected the H1299 cells with PTEN-CDS-psi-CHECK2 vector that did not contain any cognate binding consensus for miR-96-5p.

As shown in Fig. 5e, no significant change in the relative luciferase activity was detected. These data indicated that PTEN is a direct target of miR-96-5p and its binding is specifically located in 3’UTR region of PTEN mRNA. It also appeared that miR-96-5p targeting of PTEN mRNA does not occur through the binding to PTEN CDS (Fig. 5e). Next, to further confirm these data and the involvement of miR-96-5p expression in the control of PI3K-AKT pathway activation by PTEN, we evaluated by western blot analysis, the expression level of PTEN, pAKT S473 and AKT proteins in the Cal 27 cells transiently transfected with miR-96-5p inhibitor or control inhibitor. The efficiency of transfection in Cal 27 was detected by RT-qPCR (Fig. 5f). As shown in Fig. 5g, by reducing the ectopic expression of miR-96-5p, we observed an increase in the protein level of PTEN, a decrease in the expression level of pAKT S473 and no change in the expression level of AKT. These data confirm that miR-96-5p can directly target PTEN and affects PI3K/AKT pathway. The inverse correlation between the expression level of PTEN protein and miR-96-5p level was confirmed also in FaDu cell line (Additional file 5: Figure S3).

### PTEN depletion recapitulates the biological effects of miR-96-5p overexpression in HNSCC cells

**Fig. 6:**
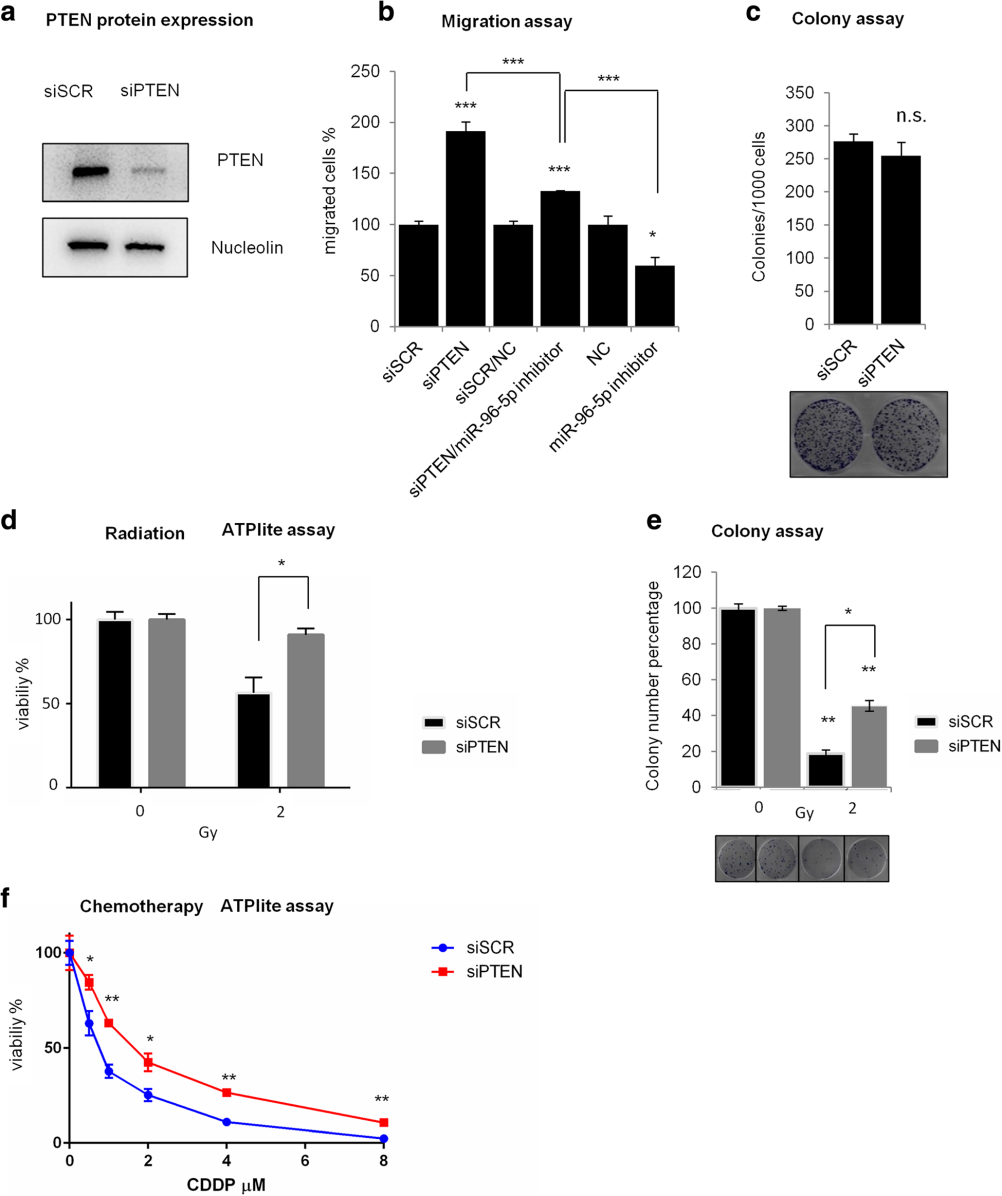
PTEN silencing mimics the biological effects of miR-96-5p overexpression in Cal 27 cells**. a** Western-blot analysis of PTEN protein expression level in Cal 27 cells transfected with siPTEN and scrambled siRNA as negative control. **b** Representative histogram of transwell migration assay of Cal 27 cells treated with siPTEN and scrambled siRNA and co-transfected cells with miR-96-5p inhibitor and siPTEN or control inhibitor and scrambeld siRNA. Bars show the means of at least two experiments performed in triplicate. The *p*-value refers to matched control inhibitor and scrambled siRNA versus miR-96-5p inhibitor and siPTEN transfected samples.*P < 0.05; **P < 0.001; ***P < 0.0001. **c** Colony formation assay of Cal 27 cells treated with siPTEN and scrambled siRNA. Histogram shows the number of migrated cells after using siPTEN and scrambled siRNA. Bars show the means of at least two experiments performed in triplicate. The p-value refers to matched control versus siPTEN transfected samples.*P < 0.05; **P < 0.001; ***P < 0.001. **d-e** Cell viability (**d**) and cell colony (**e**) analyses of Cal 27 cells transfected with siPTEN and treated with radiation at the dose of 2Gy. **f** Cell viability analysis of Cal 27 cells transfected with siPTEN and treated with different concentration of cisplatin in comparison with the negative control. siSCR: SCR siRNA, siPTEN: PTEN siRNA

To investigate whether miR-96-5p may promote cell migration and cell resistance to therapy by silencing the PTEN expression in HNSCC cells, we assessed the effect of PTEN depletion on cell migration, clonogenicity and sensitivity to radiotherapy by the transfection of PTEN siRNA for 48 h in Cal 27 cell line. The efficient knockdown of PTEN mRNA in Cal 27 cells is shown in Fig. 6a. By performing migration assay in Cal 27 cells depleted for PTEN, we observed a significant increase in the cell migration similar to miR-96-5p overexpression (Fig. 6b). To explore whether miR-96-5p regulation of cell migration is executed in a PTEN-dependent manner, we co-transfected cells with miR-96-5p inhibitor and siRNA of PTEN (siPTEN). As shown in Fig. 6b, compared with cells transfected with the relative negative control (si-scr/NC), the cells co-transfected with miR-96-5p inhibitor and siPTEN exhibited a lower increase of cell migration respect the only siRNA of PTEN (Fig. 6b), demonstrating that siRNA mediated knockdown of PTEN could partially rescue the inhibitory effect of miR-96-5p inhibitor for the cell migration. We next explored the role of PTEN silencing in the colony formation ability and in the sensitivity to radiotherapy. As expected and shown in Fig. 6c, no significant change in colonies forming between control and siPTEN transfected cells has been detected in Cal 27 cells. On the contrary, we observed an effect on cell sensitivity to radiotherapy; after 48 h of transfection with siRNA of PTEN, Cal 27 cells were treated with 2Gy dose of irradiation and the cell viability was assessed by ATPlite assay (Fig. 6d). As shown in Fig. 6 d, down-regulation of PTEN increased the cell radio-resistance. We further confirmed these data by performing a colony formation assay. As shown in Fig. 6e, a lower expression of PTEN led to an increase of colonies number formed in the cells treated with 2Gy in comparison with the negative control. Interestingly, depletion of PTEN expression rendered Cal27 cells less prone to Cisplatin-induced cell killing (Fig. 6f). Collectively these findings clearly describe that PTEN is a mediator of chemo-radiotherapy treatment for HNSCC cells and that its miR-96-5p-mediated depletion contributes to HNSCC radio-chemoresistance.

## Discussion

Despite advances in treatments, the overall mortality of HNSCC has remained unchanged over the past decades. The lethality of HNSCC is mainly due to difficulties in detecting it at an early stage and the lack of effective treatments for patients in advanced stages. Therefore, it is crucial understanding the molecular mechanisms involved in HNSCC progression and discovering more effective biomarkers of HNSCC to improve diagnosis, treatment choice and prevention of the disease.

Among the best promising biomarkers, miRNAs, are emerging as an appealing tool for screening, diagnosis, prognosis, monitoring tumor progression, assisting in designing the proper treatment for patients and predicting drug response [18]. In addition, the correction of cellular miRNA expression levels might emerge as a potential therapeutic strategy [32, 33]. We previously showed that altered expression of 12 microRNA *TP53*-dependent signature in tumors and 4 microRNA signature in matched peritumoral tissues of HNSCC are able to predict local recurrence in patients [14, 24]; both of these signatures include miR-96-5p. In addition, this miR may also work as an independent prognostic factor for local recurrence development specifically in the oral cancer subgroup of HNSCC [15] and it was identified among a pool of miRNAs, which is able to predict malignant transformation in oral leukoplakia [22]. Several studies have shown that miR-96-5p could act as an oncogene [34–38] or tumor suppressor [39, 40] according to different kinds of cancer. In HNSCC, there is short evidence showing its ability to work as an oncogene. Chung-Hsien Chou BS et al. showed that concordant up-regulation of miR-31, miR-96, and miR-182 may increase migration and invasion of HNSCC cells by co-targeting NUMB mRNA [41], while Yan Guo et al. demonstrated the involvement of miR-96 in the Tongue Cancer tumorigenesis by promoting the repression of MTSS1 mRNA expression [42].

However, no evidences about the role of miR-96-5p in chemo/radio-resistance of HNSCC have been shown yet and in this study, we provided a novel molecular insight of miR-96-5p impacting HNSCC by suppressing PTEN expression. In particular, as miR-96-5p expression was associated with *TP53* status [14], we assessed its activity specifically on HNSCC cells carrying mutant p53, which is the most frequently mutated gene in HNSCC (about 80% in HPV negative HNSCC patients from TCGA cohort).

We showed that miR-96-5p was up-regulated in tumors versus normal tissues from two different HNSCC cohorts and that its deregulation was significantly stronger in a mutant p53 context. In addition, up-regulation of miR-96-5p may promote cell migration and resistance to chemo and radio-therapy without affecting cell proliferation and clonogenicity. Cook et al., recently reported that gain of function mutp53 carrying colon cancer cells released miR-1246 enriched exosomes which instructed tumor associated macrophages toward cancer progression and metastasis [43]. This suggests that the analysis of exosomal circulating miRNAs, such as miR-96-5p, might provide additional insights regarding the molecular events underlying relapse of HNSCC patients.

**Fig. 7:**
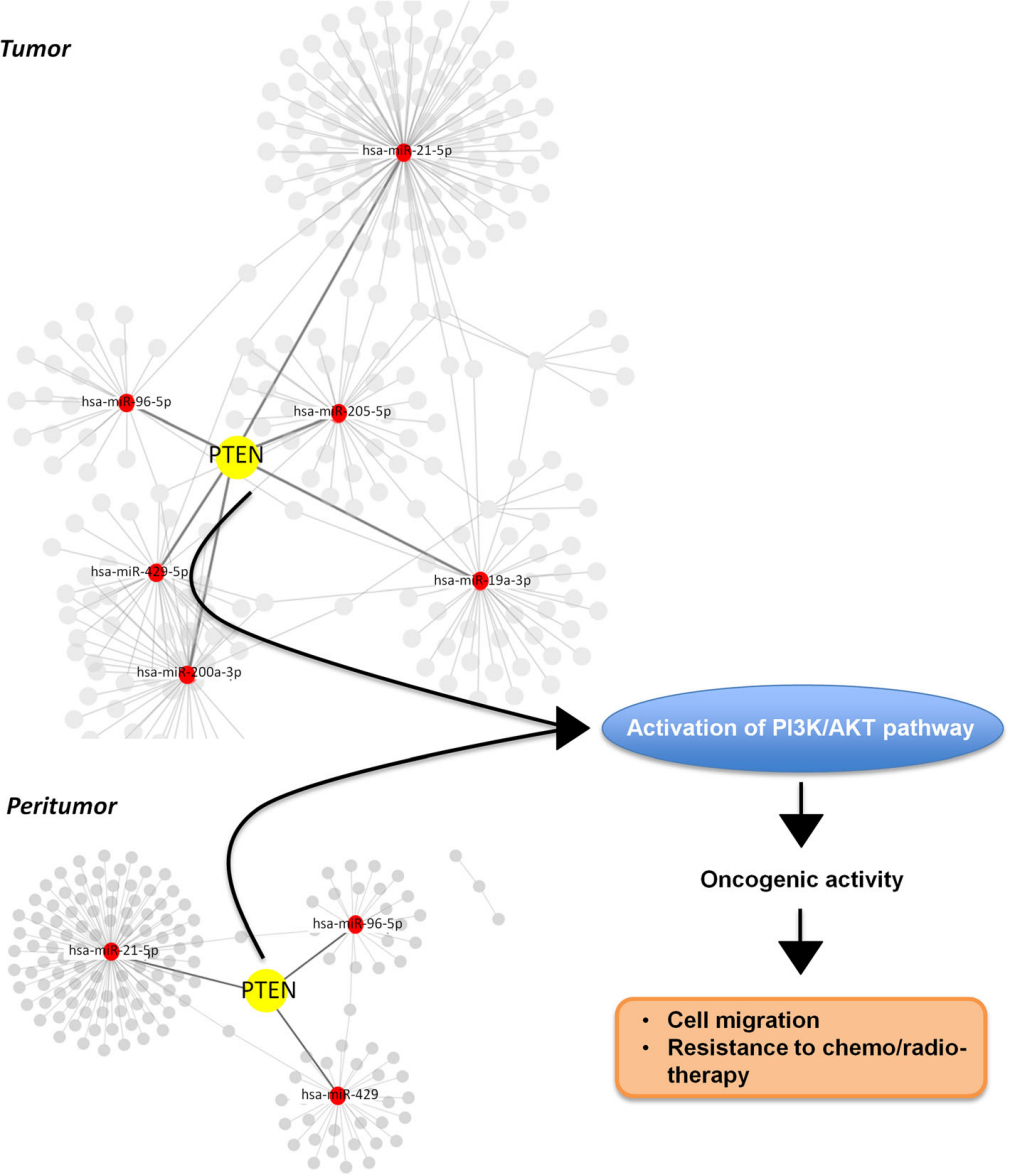
Proposed model depicting the oncogenic effects of the miR-96-5p/PTEN alterated expression in HNSCC . As shown in the miRNAs-validated targets interaction networks, PTEN is a direct target shared by both miRNA signatures in the tumoral and peritumoral HNSCC tissues. In particular, the oncogenic miR-96-5p directly binds to PTEN 3’UTR then commences the signaling cascade and leads to down regulation of the expression level of PTEN therefore, cell migration and resistance to radiotherapy and chemotherapy increase

Additionally, by the pathway enrichment analysis performed using the list of mRNAs negatively and positively correlated to miR-96-5p expression in the 176 HNSCCs carrying mutant p53 from TCGA cohort, we identified PI3K/AKT signaling pathway as one of the most significant pathways linked to miR-96-5p molecular activity. It is the most frequently altered oncogenic pathway in HNSCC playing a crucial role in regulating a broad range of cellular functions including cell migration, chemo-resistance, and radio-resistance [28–30]. In addition and more interestingly, several evidences suggest that PI3K pathway is a promising therapeutic target in HNSCC [44, 45]. Its activation is negatively regulated by a lipid phosphatase, phosphatase and tensin homolog (PTEN), which catalyzes the dephosphorylation of PIP3 to PIP2 and governs numerous cellular processes [46]. Down-regulation of PTEN expression due to either genetic alterations such as mutations and deletions or epigenetic inactivation accounts for around 15% of TGCA HNSCC patients. Here, we propose that the tight targeting of PTEN expression through aberrant expression of miRNAs including miR-96-5p might occur in the peritumoral tissues that are histologically undistinguished from matched non-tumoral tissues thereby suggesting PTEN reduced expression as an early event in HNSCC tumorigenesis (Fig. 7). This miRNA-mediated downregulation occurs also in tumoral tissues in which the number of the miRNAs (including miR96-5p) targeting directly PTEN is higher than those involved in peritumoral tissues. In aggregate these findings might suggest that persistent reduction of PTEN expression due to aberrant modulation of miRNAs is necessary for early and late stages of HNSCC tumorigenesis.

To summarize and as shown in our hypothetical model (Fig. 7), our study provided the first evidence that miR-96-5p played a significant role in enhancing resistance to therapy through inhibition of PTEN in HNSCC. Despite that, there might be other targets of miR-96-5p, which could also affect the sensitivity to chemo and radiotherapy, also in wild type *TP53* context.

Therefore, future studies are required to identify additional targets and pathways of miR-96-5p and its correlation with target therapies ongoing in clinical trial for HNSCC treatment, as PI3K inhibitors.

## Conclusions

Taken together, these results indicate that miR-96-5p could be a promising biomarker to monitor treatment response in HNSCC and identify patients resistant to chemo-radiotherapy. In addition, therapeutic strategy targeting miR-96-5p levels could potentially be beneficial to overcome chemo/radio-resistance in HNSCC.

## Additional file

Additional file 1: **Table S1** List of genes positively and negatively correlated to miR-96-5p expression in the subset of TCGA HNSCC tumors carrying missense TP53 mutations by bioinformatics analyses. (XLSX 26 kb)

Additional file 2: Supplementary material and methods. Cell cycle analysis. (DOCX 125 kb)

Additional file 3: **Figure S1.** miR-96-5p doesn’t affect cell proliferation and clonogenicity. (TIF 967 kb)

Additional file 4: **Figure S2.** miR-96-5p expression affects chemotherapy sensitivity of FaDu cells. (TIF 817 kb)

Additional file 5: **Figure S3.** PTEN protein expression increases after the transfection of miR-96-5p inhibitor in FaDu cells. (TIF 580 kb)

## Abbreviations

3’UTR: 3′ untranslated region
AKT: AKT serine/threonine kinase
CDS: coding sequence
HNSCC: Head and neck squamous cell carcinoma
miRNA: microRNA
mTOR: The mammalian target of rapamycin
N: norma1
n.s: not significant
PI3K: Phosphatidylinositol-4 5-bisphosphate 3-kinase
PTEN: Phosphatase and tensin homolog
siRNA: small interfering RNA
T: Tumor
TCGA: The Cancer Genome Atlas
