# Supplementary material for: miR-96-5p targets PTEN expression affecting radio-chemosensitivity of HNSCC cells
Source: J Exp Clin Cancer Res. 2019 Mar 29;38:141. doi: 10.1186/s13046-019-1119-x (PMC6440033; doi:10.1186/s13046-019-1119-x)
Supplement: Supplementary file 2 — Supplementary material and methods. Cell cycle analysis. (DOCX 125 kb) [file 13046_2019_1119_MOESM2_ESM.docx]

**Additional file 2: Supplementary material and methods**

**Cell cycle analysis.**

Cal 27 and FaDu cell lines were transfected with mirVana™ miRNA inhibitor Negative Control #1 (Ambion) and hsa-miR-96-5p mirVana™ miRNA inhibitor (Ambion # 4464084). Cells were harvested, washed with PBS1x, resuspended in PBS1x/EDTA 5mM, fixed in 70% ETOH/PBS1x. Fixed cells were treated with RNase at final concentration of 1 mg/ml for 30 min at 37°C before addition of 5 mg/ml propidium iodide (PI) (Sigma) and analyzed by FACS.

**Additional file 3: Figure S1. miR-96-5p doesn’t affect cell proliferation and clonogenicity.** Two HNSCC cell lines (Cal 27, FaDu) were transfected with miR-96-5p inhibitor. **a-d)** The histogram shows efficiency of transfection and the number of colonies formed by negative control (NC) and miR-96-5p transfected Cal 27 **(a-b)** and FaDu **(c-d)** cell lines. **(e-f)** Growth curve of Negative control and miR-96-5p inhibitor transfected Cal 27 **(e)** and FaDu **(f)** cells assessed by Trypan blue staining. The graph shows the percentage of live cells (Negative control cells/miR-96-5p inhibitor expressing cells-Y-axis) at the indicated times (x axis). **g-h)** The histograms showing the percentage of the cell cycle at 48 h after transfection. Histogram bars shows the means of at least two experiments performed in triplicate. FACS analysis of Cal 27 **(g)** and FaDu **(h)** cells transfected with miR-96-5p inhibitor.

**Additional file 4: Figure S2.** **miR-96-5p expression affects chemotherapy sensitivity of FaDu cells.** FaDu cells were transfected with miR-96-5p mimic and inhibitor. After 48 hours cells were treated with different concentration of cisplatin (0.5,1,2,4,8 μg/ml)**. a)** RT-qPCR data showing efficiency of miR-96-5p mimic transfection. **b)** Cell viability analysis of FaDu cells treated with miR-96-5p mimic and different concentration of cisplatin in comparison with the negative control. **c)** RT-qPCR data showing efficiency of miR-96-5p inhibitor transfection. **d)** Cell viability analysis of FaDu cells treated with miR-96-5p inhibitor and different concentration of cisplatin in comparison with the negative control. **e)** Colony formation assay performed on FaDu cells treated with miR-96-5p inhibitor and different concentration of cisplatin in comparison with the negative control. Histogram bars show the means of at least three experiments performed in triplicate.*P < 0.05; **P < 0.001.

**Additional file 5: Figure S3. PTEN protein expression increases after the transfection of miR-96-5p inhibitor in FaDu cells. a)** Real time graph showing miR96-5p expression level in FaDu cell line, **p value<0.001. **b)** Western-blot analysis of PTEN protein expression level in FaDu cells that were transfected with miR-96-5p inhibitor or control inhibitor
