# Supplementary figures and images for: miR-96-5p targets PTEN expression affecting radio-chemosensitivity of HNSCC cells
Source: J Exp Clin Cancer Res. 2019 Mar 29;38:141. doi: 10.1186/s13046-019-1119-x (PMC6440033; doi:10.1186/s13046-019-1119-x)

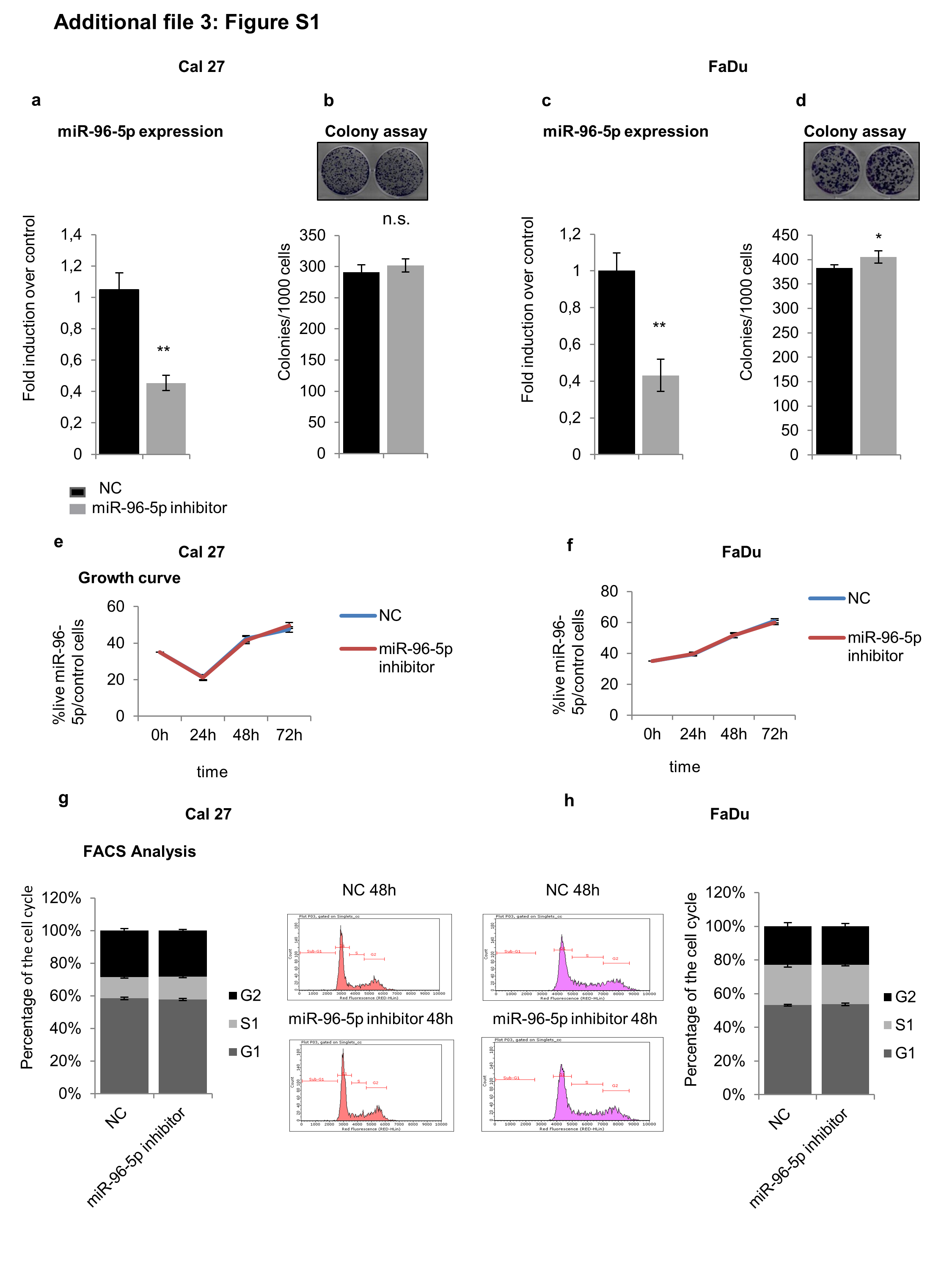

Supplement: Supplementary file 3 — Figure S1. miR-96-5p doesn’t affect cell proliferation and clonogenicity. (TIF 967 kb) [file 13046_2019_1119_MOESM3_ESM.tif]

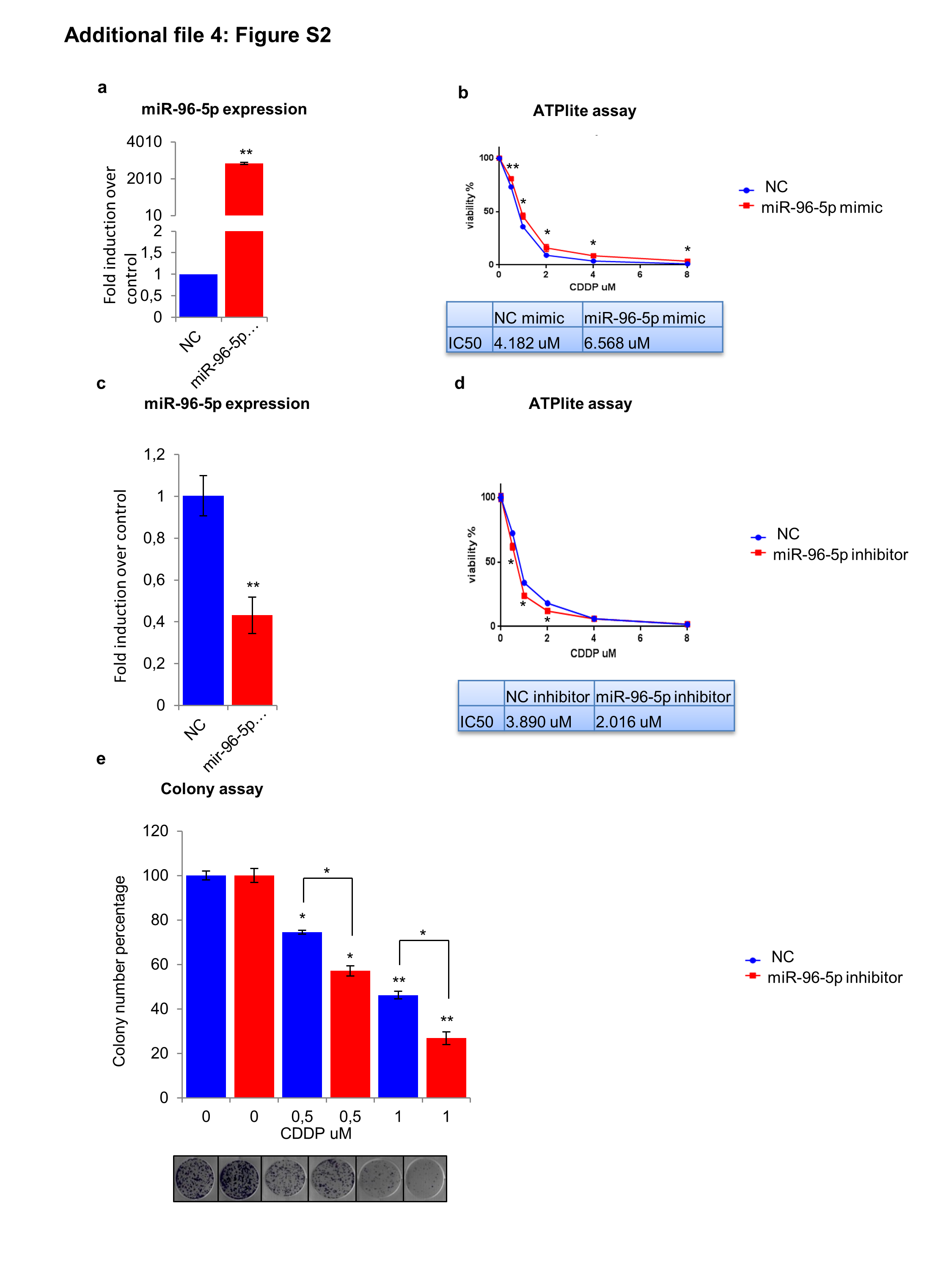

Supplement: Supplementary file 4 — Figure S2. miR-96-5p expression affects chemotherapy sensitivity of FaDu cells. (TIF 817 kb) [file 13046_2019_1119_MOESM4_ESM.tif]

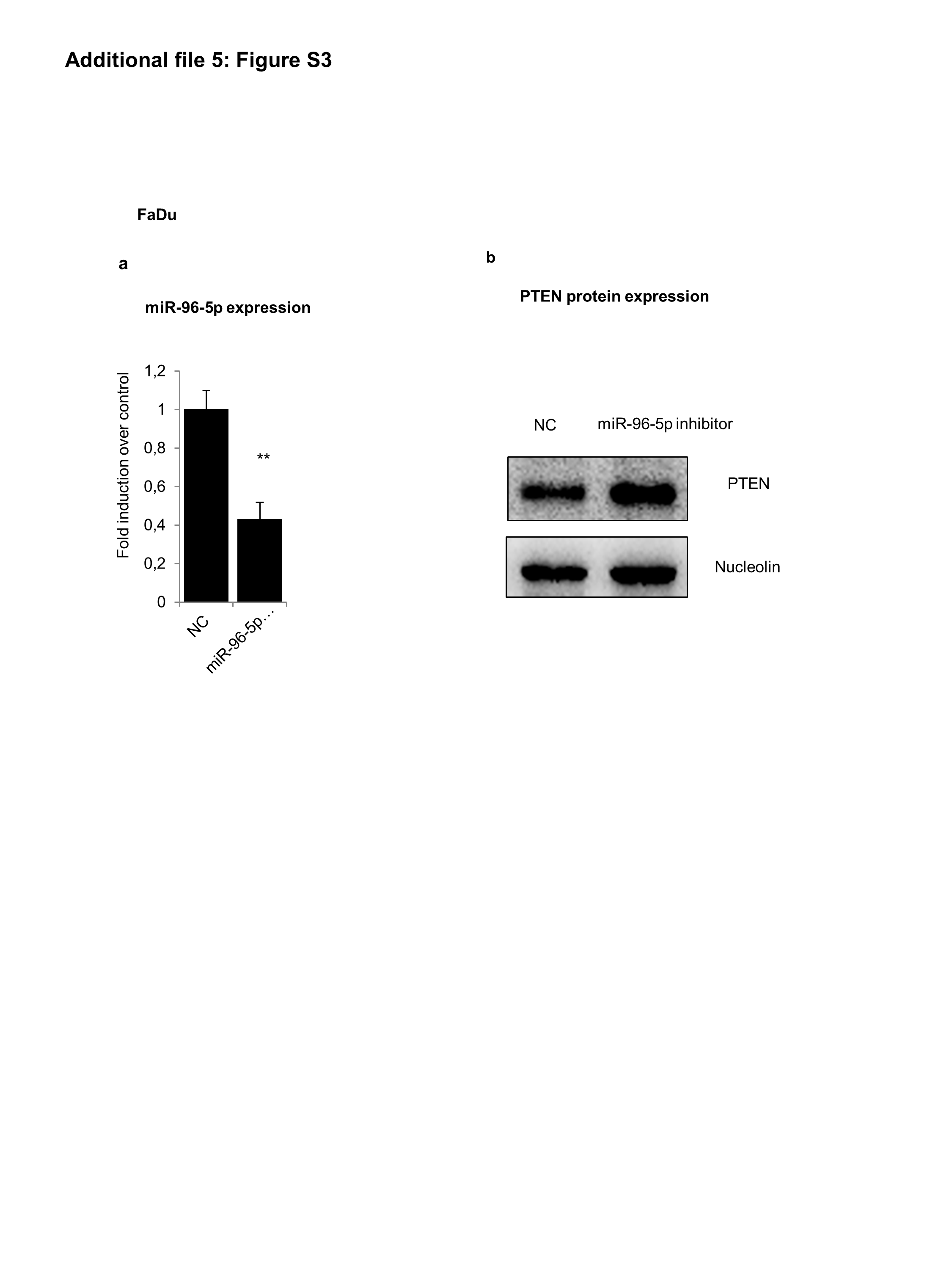

Supplement: Supplementary file 5 — Figure S3. PTEN protein expression increases after the transfection of miR-96-5p inhibitor in FaDu cells. (TIF 580 kb) [file 13046_2019_1119_MOESM5_ESM.tif]
